# Characterization and functional analysis of miRNAs in *Salvia miltiorrhiza*-isolated exosome-like nanoparticles for gastric cancer treatment

**DOI:** 10.1371/journal.pone.0353354

**Published:** 2026-07-16

**Authors:** Gang Chen, Qinjuan Zhong, Ning Tang

**Affiliations:** Department of Spleen and Gastroenterology, Shaoxing city Keqiao District Hospital of traditional Chinese Medicine, Shaoxing, Zhejiang, People’s Republic of China; Baotou Medical College, CHINA

## Abstract

**Background:**

Gastric cancer (GC) is a widespread global malignancy, frequently diagnosed at advanced stages owing to the subtle nature of early symptoms and low screening rates. Consequently, there is an imperative need to investigate innovative strategies for the treatment of GC. Exosomes, originating from various sources, act as natural nanocarriers enriched with numerous bioactive molecules, showcasing potential as groundbreaking treatments for GC.

**Materials and methods:**

In the present study, exosome-like nanoparticles (ELNs) were successfully purified from *Salvia miltiorrhiza* Bunge (Danshen) utilizing ultracentrifugation. The impact of these ELNs on human gastric cancer cells (HGC-27) was evaluated via a suite of viability assays, encompassing CCK-8, colony formation, flow cytometry, transwell migration, and wound healing assays. Furthermore, miRNA sequencing was conducted to identify and analyze the highly abundant miRNA within Danshen-derived ELNs, along with its potential biological functions.

**Results:**

The HGC-27 cells internalized Danshen-derived ELNs (DS-ELNs), which led to a decrease in cell viability. Concurrently, these ELNs exhibited significant inhibitory effects on cell migration, colony formation, and overall progression of GC in vitro. The functional analysis of the miRNAs harbored by these ELNs indicated that they may serve as a potential therapeutic target for GC.

**Conclusion:**

In summary, these findings have not only comprehensively characterized the miRNAs present in Danshen-derived exosomes but also provided invaluable insights into the molecular mechanisms by which Danshen exerts its effects in mitigating

## Introduction

Gastric carcinoma (GC), originating from the gastric epithelium, is a complex and heterogeneous malignancy influenced by the intricate interplay of environmental and genetic factors [1,2]. Based on the latest epidemiological statistics, GC ranks as the fourth leading cause of cancer-related mortality worldwide, with patients in advanced stages having a median survival rate of less than 12 months [3,4]. Despite therapeutic advancements, GC persists as a significant global health challenge owing to its pronounced invasiveness and heterogeneity [5,6]. Consequently, there is an imperative need to explore innovative therapeutic strategies for the treatment of GC.

Exosomes, constituting a significant subset of the broader category of nanoparticles secreted by various cell types, are enriched with proteins, lipids, DNA, and RNA [7]. Notably, plant-derived exosome-like nanoparticles (ELNs) have attracted increasing attention due to their biocompatibility, cost-effectiveness, environmental sustainability, and scalability. These ELNs have shown promise in the treatment of inflammatory and malignant diseases, attributed to their rich bioactive chemical content [8]. For example, ELNs derived from blueberries, garlic, ginger, and Brucea javanica have demonstrated anti-inflammatory and anti-tumor activities in various experimental models [9–13].

*Salvia miltiorrhiza* Bunge (Danshen) is a widely utilized medicinal herb in traditional Chinese medicine, recognized for its therapeutic properties, including the enhancement of blood circulation, alleviation of menstrual discomfort, and a range of pharmacological effects such as anti-tumor, cardiovascular, cerebrovascular, anti-inflammatory, and antioxidant activities [14]. Importantly, there is growing evidence that Salvia miltiorrhiza has significant anti-tumor potential. Its major bioactive components, such as salvianolic acid and tanshinones, have been extensively studied for their therapeutic effects in cancer. Among them, tanshinone IIA has attracted particular attention due to its ability to inhibit tumor cell proliferation, induce apoptosis, inhibit metastasis, and modulate the tumor microenvironment in various cancer types, including gastric cancer. Salvianolic acid, particularly salvinorin B, has also been reported to have anti-angiogenic and anti-metastatic effects, further supporting the potential of tansy as a promising candidate for cancer therapy [15].

In conclusion, plant-derived ELNs, especially those from Danshen, represent a novel and promising avenue for the development of innovative cancer therapies. Further studies are needed to elucidate their mechanism of action and therapeutic potential in gastric cancer.

## Materials and methods

### Cell culture

The human gastric carcinoma cell line HGC-27, purchased from Procell (Wuhan, China), was cultured in DMEM medium(Procell) supplemented with 10% fetal bovine serum (Hyclonge Healthcare Life Sciences, Logan, UT, USA), 100 U/ml penicillin. and 100 μg/mL streptomycin at a temperature of 37 °C in a humid atmosphere containing 5% CO2.

### Isolation and purification of ELNs

The root portion of fresh Danshen (*Salvia miltiorrhiza*) used in this study was purchased from a local agricultural market, mixed with 10 mL of phosphate-buffered saline (PBS), macerated, and the resulting juice was subjected to a series of centrifugation steps at different speeds and temperatures. Filtration is then carried out through a 100 mesh cell strainer and a filter with a pore size of 0.45 µm, with the final pellet of exosome-like nanoparticles (ELNs) being resuspended in 1 ml of sterile PBS and stored at −80° for subsequent analysis C stored.

### Nanoparticle tracking analysis (NTA)

Using a NanoSight LM20 instrument (NanoSight, Amesbury, UK), the size and number of Danshen-derived ELNs were measured in an ambient environment. The light-scattering method known as NTA was used to ascertain the particle dispersion profile. Through the use of transmission electron microscopy, the form and structure of ELNs were examined (Thermo Fisher Scientific, Waltham, MA, USA).

### RNA extraction

After extracting total RNAs from ELNs with TRIzol (Invitrogen), RNAs were purified using two 15-min phenol-chloroform treatments (Solarbio, Beijing, China). Using RQ1 DNase (Promega, Darmstadt, Germany), the obtained RNAs were subjected to DNA removal. The quality and quantity of the purified RNAs were assessed using Smartspec Plus (Bio-Rad, Hercules, CA, USA) and 1.5% agarose gel electrophoresis was used to check the integrity of the RNAs.

### Cell viability detection

GC cell proliferation was measured using the cell counting kit-8 (Beyotime, Peking, China). HGC-27 cells (5 × 10^3^ cells/well in 200 μL medium) were seeded into each well of a 96-well cell culture plate and the cells were allowed to incubate throughout the night. After adding 20 μL of CCK-8 reagent to each well at 24, 48, and 72 hour intervals, the plates were incubated for two hours. The optical density was then measured using a microplate reader (Bio-Rad, USA) at 450 nm. Five duplicates of the experimental procedures were performed for each group.

### Flow cytometric detection

Cell apoptosis was measured by flow cytometry using the Annexin V-conjugated FITC apoptosis detection kit. After 48 hours of treatment, cells were removed, washed twice with PBS, and then incubated with Annexin V-FITC and PI for 10 minutes without light. MoFLO XDP (Beckman Coulter, Brea, CA, USA) was then used to analyze the labeled cells.

### Transwell invasion

For the invasion experiment, chambers in 24-well plates with Transwell inserts with 8 μm pores (Solarbio, Beijing, China) were used. Before seeding the cells (10^5^) in the upper chamber, the inserts were coated with 50 μl of Matrigel diluted 1:4 in serum-free material. The invasive cells on the bottom of the inserts were fixed with 4% paraformaldehyde and stained with 0–1% crystal violet after incubation. Finally, a stereomicroscope (Leica, Wetzlar, Germany) was used to capture the images of the invasive cells.

### Wound healing assay

Six-well plates containing the treated cells were used. Each well was seeded with 2 × 10^5^ cells and allowed to reach confluence. After 12 hours, the confluent monolayers in each well were gently washed with PBS to remove non-adherent cells, and a linear wound was created using a sterile 50 μL pipette tip. Images of each well were captured using a Zeiss microscope with 200 × magnification (Leica, Wetzlar, Germany) at 0, 24, and 48 hours after injury to assess wound healing. The wound area was measured at each time point using ImageJ software (NIH, Bethesda, MD, USA). The migration rate was calculated using the following formula:

Migration rate (%)=Initial wound areaInitial wound width (0h)−Wound width at indicated time ×100%

### Cell clone formation assay

HGC-27 cells (500 cells/well in 2 mL medium) were seeded into six-well plates and incubated for 10–14 days. After incubation, the colonies were fixed and stained with 0.4% crystal violet (Solarbio, Beijing, China). The number of colonies was counted using an inverted microscope (Leica, Germany). For the 96-well plate assay, 1,000 HGC-27 cells were seeded per well in 200 μL of culture medium.

### MiRNA sequencing and analyses

Using the NEBNext® Multiplex Small RNA Library Prep Set for Illumina® (NEB, USA), a small RNA-cDNA library was prepared using the 3 μg of total RNAs from each sample. The Novaseq 6000 platform (Illumina Inc.) was used to sequence the libraries. USA, California). Perl and Python scripts were used to filter the raw data to eliminate low-quality reads and ensure the quality of subsequent analysis.

After mapping to the reference genome, the high-quality reads (18–30 bp) were processed using the Bowtie program [16].To find known miRNAs, the accurately mapped reads were compared with the miRBase22.0 database. MiRNA counts were calculated using the MirDeep2 quantifier. pl [17].The base bias for each position of each detected miRNA as well as the original location of a found miRNA with a specific length were calculated using custom scripts. To normalize the miRNA expression levels, the TPM (Transcript per Million) was used [18].

Using Miranda3.3 software and RNAhybrid, target genes were identified using the human genome as a reference genome [19,20]. The program mentioned above examines how complementary miRNA and the 3’UTR region are. To arrive at the final result, which is the weighted sum of the base pair and gap match and the mismatch values, the binding energy of the duplex structure, the evolutionary conservation of the entire target site and its position in the 3’UTR are all required to be calculated and integrated [21]. The following parameters were set to analyze potential target genes and binding sites of the candidate miRNAs: -sc 140 -en-10 -scale 4-strict; −10 -p-value_cutoff 0.05 -m max_target_length 50,000.

### Statistical analyses

In the current study, statistical significance was determined using Microsoft Excel (2021). The mean±standard deviation was used when reporting data. When comparing the means of two groups of data, statistical significance was ensured using Student’s t-test (paired). p-values less than 0.05 indicate statistically significant differences.

### Online data deposition

The National Center for Biotechnology Information accession number for the datasets used in this study is PRJNA1136078.

**Consent:** This study does not involve human beings or animals as subjects, and it uses the published and commercialized HGC-27 cell line, so it usually does not obtained the approval of the ethics Committee.

## Results

### Isolation and characterization of Danshen-derived ELNs

**Fig 1:**
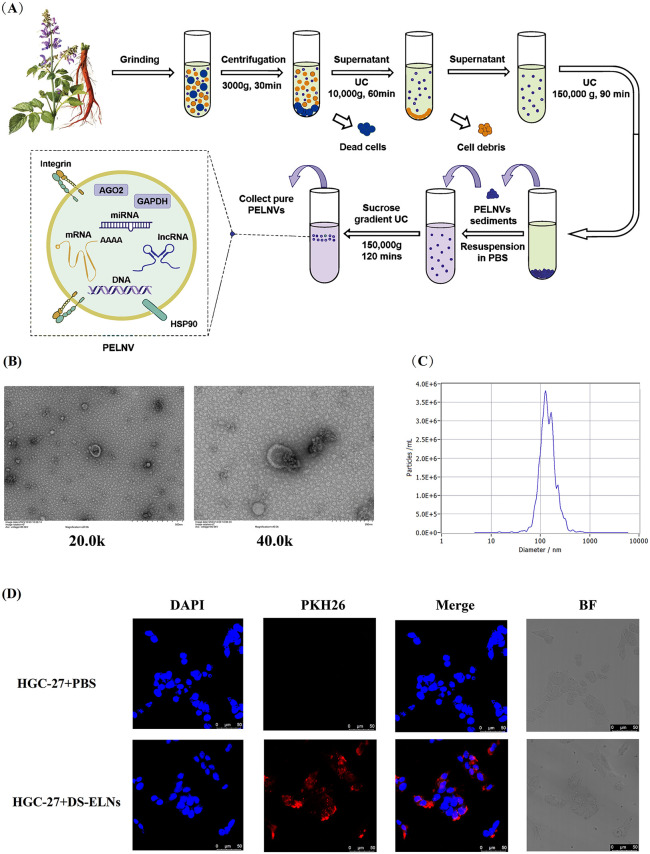
Characterization and cellular internalization of ELNs derived from Danshen. **(A)** The schematic of the isolation and purification methods of Danshen-derived ELNs. **(B)** The size of ELNs were analysed by NTA. **(C)** Transmission electron microscopy (TEM) image of ELNs (scale bar = 100 nm). **(D)** The PKH26 (in red)-labeled ELNs were taken up by HGC-27 cell, while cell nucleus were stained with DAPI (blue). Scale bar = 25 μm.

The ELNs were extracted and refined from freshly harvested Danshen, by utilizing methods of differential centrifugation and ultracentrifugation (Fig 1A). Through TEM and NTA, the putative ELNs were thoroughly characterized. It was revealed that the ELNs exhibited a typical cup- or spherical-shape structure, with an average size approximately 150.8 nm (Fig 1B and 1C), indicating the successful isolation of ELNs from Danshen.

To explore the biological activity of ELNs *in vitro*, it is critical to ensure that Danshen-derived ELNs are properly internalized by cells. ELNs were tagged with PKH26, a lipophilic fluorescent dye, and then incubated with HGC-27 cells at 37 °C for 24 h. The cells were then stained with the nuclear DNA dye DAPI and photographed with a confocal microscope. The results indicated that the red fluorescent signal of the ELNs group could be seen in the cell cytoplasm, but not in control tests (Fig 1D), showing that ELNs generated from Danshen could be absorbed successfully by GC cells.

### Danshen-derived ELNs regulate the proliferation and apoptosis of GC cells

**Fig 2:**
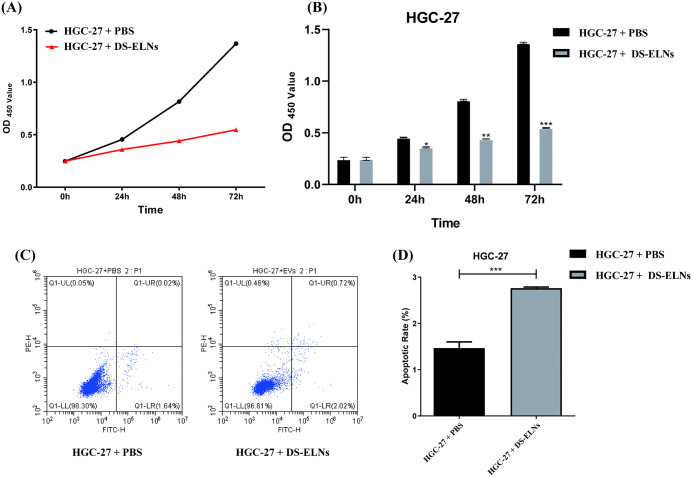
Danshen-derived ELNs regulate the proliferation and apoptosis of HGC-27 Cells. **(A)** The proliferation of the gastric cancer cells treatment with ELNs was detected by CCK-8 assays. **(A)** Representative images and **(B)** quantitative statistics of plate clone formation assay. **(C)** Flow cytometry results and **(D)** quantitative statistics of tumor cell apoptosis.

The impact of Danshen-derived ELNs on cell viability were determined using the CCK-8 test. In the CCK-8 experiment, HGC-27 cells were treated with ELNs (10^9^ particles/ml) and PBS. After incubation for 24, 48, and 72 hours, the findings showed that ELNs therapy may inhibit the growth of cancer cells (Fig 2A and 2B) as compared to PBS treatment. The colony formation test was conducted. The picture of crystal violet staining and quantification demonstrated that ELN therapy could considerably limit growth of GC cell (Fig 2C and 2D).

Furthermore, flow cytometry was used to evaluate the anti-tumor efficacy of Danshen-derived ELNs. The GC cell line was treated with ELNs (10^9^ particles/ml) and PBS for 24 h before incubating with Annexin V-FITC/PI. Annexin V-FITC stained early cell apoptosis, while PI identified late-death cells. Finally, the number of stained cells was determined using a flow cytometer, which revealed that ELNs might enhance cell death in contrast to the PBS group (Fig 2E and 2F). In conclusion, Danshen-derived ELNs exert anti-tumor actions via regulating proliferation and apoptosis of GC cells.

### Danshen-derived ELNs mediate the migratory potential of GC cells

**Fig 3:**
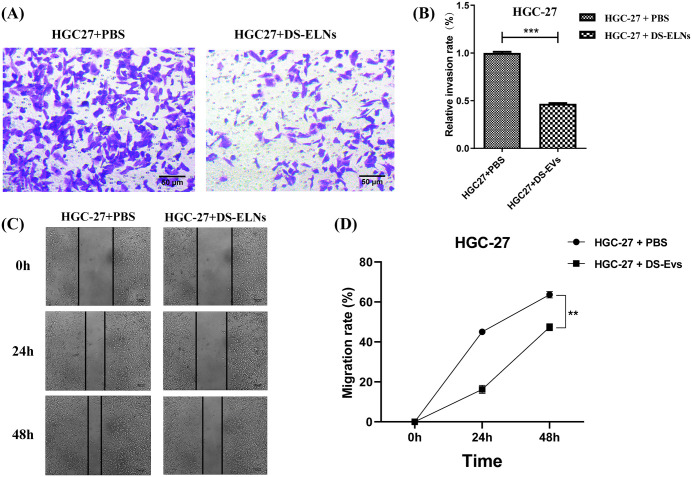
Migration inhibition effects of Danshen-derived ELNs. **(A)** wound healing images and **(B)** quantitative statistics of HGC-27 cell treat with or without ELNs-treatment. **(C)** Transwell migration images and **(D)** quantitative statistics of HGC-27 cell treat with or without ELNs-treatment.

The transwell migration experiment was used to investigate whether Danshen-derived ELNs can control the GC cells’ ability to migrate. First, 10^9^ particles/ml of serum-free medium were introduced to the upper chamber containing ELNs, and the cells were grown there for a full day. Cells will migrate lower over time to the rich nutrient medium. The number of GC cells that migrated under a microscope was then counted, and the results showed that there was a notable decrease in the number of invasive cells after ELN therapy as compared to control cells. (Fig 3A and 3B), indicating that Danshen-derived ELNs may considerably impede HGC-27 cell migration in vitro.

The purpose of the wound healing experiment was to evaluate the GC cells’ ability to migrate after receiving ELNs. The results, when compared to the normal control, demonstrated that ELNs suppressed cell invasion for 24 and 48 hours after treatment (Fig 3C and 3D).

### The Enriched miRNAs of Danshen-derived ELNs

To investigate the functional mechanism of Danshen-derived ELNs, the ELNs samples extracted above were sequenced using miRNA-seq. Three libraries (Exo-1, Exo-2, and Exo-3) were successfully created and examined. Sequencing quality control findings demonstrate that the Q20 and Q30 of the off-line sequencing sequence are both 80%, indicating that these data sets are highly repeatable and trustworthy, and may be utilized for further research (S1 Table).

**Fig 4:**
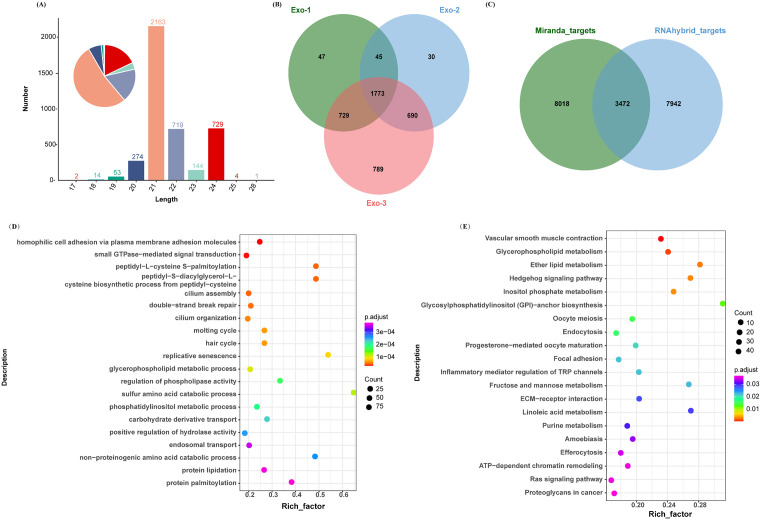
The miRNA identification and functional analysis of its target genes. **(A)** The length distribution of the exosomes miRNA from Danshen. **(B)** Venn diagrams of miRNAs identified from 3 groups of Danshen-derived exosomes. **(C)**Target genes of candidate miRNA identified based on Miranda3.3 software and RNAhybrid software. **(D)** GO enrichment analysis of target genes predicted by highly enriched miRNAs in Danshen-derived exosomes **(E)** KEGG enrichment analysis of target genes predicted by highly enriched miRNAs in Danshen-derived exosomes.

A total of 4,103 miRNAs were identified from the ELNs of Danshen (S2 Table). Through further analysis of the data, it was found that the length of these 4,103 miRNAs was in the range of 17–28pb, and the sequence with the length of 21pb was the most, accounting for 52.72% (2,163) (Fig 4A and S3 Table). At the same time, it was also found that 1,773 miRNAs were identified in these three biological repetitions (Fig 4B).

### Functional analyses of target genes for miRNAs Danshen-derived ELNs

To explore the potential molecular effectors of ELNs from danshen in suppressing the formation and progression of gastric cancer, we used Miranda3.3, and RNAhybrid software to predict the target genes of 287 miRNAs (The original reads in each biological repetition is not less than 10.) identified above. A total of 3472 target genes were identified (S4 Table).

In order to investigate the main functions involved in the predicted target genes, KEGG analysis was carried out (Fig 4E), and 25 functional pathways were found (P < 0.01) (S4 Table). It showed that multiple enriched pathways are related to inflammation, promoting blood circulation and regulating menstruation, removing blood stasis and relieving pain and tumor, including “Vascular smooth muscle contraction” (ID: hsa04270), “Glycerophospholipid metabolism” (ID: hsa00564), “Hedgehog signaling pathway”(ID: hsa04340), “Oocyte meiosis”(ID: hsa04114),” Progesterone-mediated oocyte maturation” (ID: hsa04914), “Focal adhesion” (ID: hsa04510), “Inflammatory mediator regulation of TRP channels”(ID: hsa04510), “Efferocytosis”(ID: hsa04148), “Ras signaling pathway” (ID: hsa04014), “Proteoglycans in cancer” (ID: hsa05205) (Fig 4E and S5 Table). It suggested that the highly enriched miRNAs of Danshen-derived ELNs might play an important role in cross-kingdom regulation.

In addition, GO analysis was performed to analyze the major functions involved in the predicted target genes. It also defines several rich terms closely related to biological processes such as tissue repair and cell metabolism, including “homophilic cell adhesion via plasma membrane adhesion molecules”(GO:0007156), “small GTPase-mediated signal transduction”(GO:0007264), “cilium organization” (GO:0044782), “molting cycle” (GO:0042303), “replicative senescence” (GO:0090399), “glycerophospholipid metabolic process” (GO:0006650), “sulfur amino acid catabolic process”(GO:0000098), “phosphatidylinositol metabolic process”(GO:0046488) and other terms (Fig 4D and S6 Table). These results suggest that Danshen-derived ELNs may regulate the proliferative and migratory potentials of HGC-27 cells through signaling.

## Discussion

Cancer is one of the leading causes of mortality worldwide, despite society’s ongoing development and advancement. As a result, it is a novel approach to cancer prevention and treatment by discovering new and effective anticancer drugs from natural sources [22,23]. Recently, more and more studies support the idea that many successful anticancer drugs that are in clinical use and have demonstrated significant efficacy are derived from natural bioactive components as medicinal plants [24,25]. For instance, Dendrobium plants have demonstrated anti-tumor properties, primarily attributed to their active components such as polysaccharides, alkaloids, phenanthrenes, bibenzenes, and fluorenones [26].

Danshen, a traditional Chinese herbal medicine, plays a significant role in the treatment of various clinical diseases, including cardiovascular and gastrointestinal disorders. Previous studies have shown that Tanshinone IIA (Tan IIA), a pharmacologically active component extracted from Danshen, can inhibit the proliferation of gastric cancer (GC) cells by inducing ferroptosis mediated by p53 upregulation [27]. Similarly, Salvianolic acid F (SalF) from Danshen has been reported to suppress lung cancer cell growth by activating apoptotic signaling pathways and inhibiting anti-apoptotic gene expression [28].

In our current study, we found for the first time that Danshen-derived extracellular vesicle-like nanoparticles (ELNs) could suppress the proliferation of GC cells in vitro, suggesting a potential anti-cancer role for Danshen ELNs. This finding is consistent with previous reports on plant-derived ELNs, such as those from ginger and grapes, which have also demonstrated anti-tumor effects through the delivery of bioactive molecules [29,30]. However, compared to these studies, our work provides novel insights into the specific miRNA cargo of Danshen ELNs and their potential mechanisms of action.

Exosome is type of extracellular vesicles, which are nanostructures formed by the fusion of vesicles and plasma membrane, and contain various bioactive compounds such as lipids, DNA, proteins, and miRNAs [31,32]. Increasing evidence indicates that plant ELNs-derived miRNAs have the ability to resist external environmental degradation, cross the gastrointestinal barrier, and have regulatory effects in mammals after ingestion, such as anti-inflammatory, anti-virus and anti-tumor, which reveals that plant miRNAs have high application potential in RNA-based molecular therapy and the development of new therapeutic drugs [33]. In the current study, the isolation and purification of ELNs of Danshen, was performed successfully. In addition, the potential anti-cancer mechanism of Danshen ELNs was analyzed by miRNA sequencing experiment. A total of 1773 common miRNAs were identified in the exosomes of danshen, and 287 miRNAs (S4 Table) were identified in three biological repeats, which indicated that the miRNAs enriched in ELNs may play a role through some unknown mechanism.

Notably, our analysis revealed that miR4103, miR4102, and miR4101 are the most abundant miRNAs in Danshen-ELNs (S4 Table). Functional annotation demonstrates that miR4102 is enriched in core biological processes including metabolic regulation (e.g., FoxO and insulin signaling pathways), DNA repair (via non-homologous end-joining), cardiovascular homeostasis (linked to relaxin signaling), cancer progression (gastric cancer pathways), and cellular senescence (S1A–S1C Fig; S7 Table). These findings align with the multifaceted roles of miRNAs in cross-kingdom regulation. Furthermore, bioinformatics prediction further identifies CDK2, a central regulator of the cell cycle, as a potential target of these miRNAs in human gastric cancer cells. Given that CDK2 activation drives G1/S transition and cell proliferation, its suppression by Danshen ELN-derived miRNAs may inhibit gastric cancer growth through FoxO-mediated cell cycle arrest (via downregulating cyclin-dependent kinases) and senescence-associated tumor suppression (by activating p53/p21 pathways). This mechanism is consistent with prior studies showing plant miRNAs (e.g., from ginger and broccoli) modulate mammalian gene networks to impede tumorigenesis [34,35]. Our work extends these observations by pinpointing specific miRNAs from Salvia miltiorrhiza ELNs and their predicted targets in gastric cancer. To further elucidate the potential mechanisms, we propose that Danshen ELNs-derived miRNAs may exert their anticancer effects through several pathways. First, miR4102 could directly target and degrade mRNAs of oncogenes or cell cycle regulators, such as CDK2, thereby disrupting cell cycle progression and synergizing with FoxO signaling to induce apoptosis or senescence [36]. Second, these miRNAs may activate tumor suppressor pathways, such as the p53 pathway, which is known to be involved in promoting DNA repair (via homologous recombination/NHEJ crosstalk) or amplifying cellular senescence signals through SASP (senescence-associated secretory phenotype) modulation [37]. Additionally, the anti-inflammatory properties of these miRNAs could contribute to the overall anticancer effect by suppressing NF-κB-driven inflammation (a hallmark of adipocytokine signaling dysregulation), thereby remodeling the tumor microenvironment and attenuating chronic inflammation, which is a known risk factor for cancer development [38].

In conclusion, this current study provides evidence that Danshen ELNs-derived miRNAs may inhibit the progression of GC by targeting key genes such as CDK2 and modulating cell cycle and apoptosis-related signaling pathways, thereby mediating cross-species gene regulation. These findings not only provide a stronger theoretical foundation for the potential anti-cancer application of *Salvia miltiorrhiza*, but also offer new perspectives for the development of plant-based anti-cancer therapeutics. Future studies should aim to experimentally validate the direct targets of these miRNAs, elucidate the underlying signaling mechanisms, and evaluate the in vivo efficacy of Danshen ELNs in cancer treatment.

## Supporting information

S1 Fig Target gene predicted by miR4102 from Danshen-derived exosomes and its KEGG enrichment analysis. (A) Target gene predicted by miR4102 from Danshen-derived exosomes. (B-C) The KEGG enrichment analysis of miR4102 from Danshen-derived exosomes.
(TIF)

S1 Table Quality of quality control of danshen-derived exosomal miRNA-seq data.csv. (CSV)

S2 Table List of identified miRNAs in danshen-derived exosomes.csv. (CSV)

S3 Table The length distribution of identified miRNAs in danshen-derived exosome.csv. (CSV)

S4 Table Prediction of target genes of miRNAs from Danshen-derived exosome.xlsx. (XLSX)

S5 Table KEGG enrichment analysis of target genes predicted by highly enriched miRNAs in Danshen-derived exosomes.csv. (CSV)

S6 Table GO enrichment analysis of target genes predicted by highly enriched miRNAs in Danshen-derived exosomes.csv. (CSV)

S7 Table Target gene predicted by miR4102 from Danshen-derived exosomes and its KEGG enrichment analysis.xlsx. (XLSX)
