# Supplementary material for: Characterization and functional analysis of miRNAs in Salvia miltiorrhiza-isolated exosome-like nanoparticles for gastric cancer treatment
Source: PLoS One. 2026 Jul 16;21(7):e0353354. doi: 10.1371/journal.pone.0353354 (PMC13375030; doi:10.1371/journal.pone.0353354)
Supplement: Attachment — Submitted filename: Response to Reviewers.docx [file pone.0353354.s011.docx]

Characterization and Functional Analysis of miRNAs in Salvia miltiorrhiza-Isolated Exosome-Like Nanoparticles for Gastric Cancer Treatment

**Review Comments to the Author**

Please use the space provided to explain your answers to the questions above. You may also include additional comments for the author, including concerns about dual publication, research ethics, or publication ethics. (Please upload your review as an attachment if it exceeds 20,000 characters).

**Reviewer #1:** **1)Firstly，multiple active components in Salvia miltiorrhiza have antitumor effects on gastric cancer, such as salvianolic acid B, tanshinone I, tanshinone IIA, etc. They exert their curative effects by regulating multiple molecular targets, such as cell cycle regulatory proteins and apoptosis-related proteins. The anti-gastric cancer effect of Salvia miltiorrhiza has been preliminarily confirmed in clinical and animal experiments. However, due to the lack of large-sample and multi-center clinical studies, its status in the treatment of gastric cancer still needs to be further determined.**

**Respond to Reviewer :**Thank you very much for your careful review of our manuscript and your valuable comments. We fully agree with your opinion regarding the antitumor effects of multiple active components in Salvia miltiorrhiza on gastric cancer. As you pointed out, although several clinical and animal studies have preliminarily confirmed that Salvia miltiorrhiza and its active components (such as salvianolic acid B, tanshinone I, and tanshinone IIA) exert antitumor effects by regulating multiple molecular targets, including cell cycle regulatory proteins and apoptosis-related proteins, the exact status of Salvia miltiorrhiza in the treatment of gastric cancer still needs to be further determined due to the lack of large-sample and multi-center clinical studies.

We sincerely appreciate your professional suggestions, which are of great significance for guiding our future research. In our subsequent work, we will pay close attention to and actively promote large-scale, multi-center clinical studies in this field, in order to provide more solid evidence for the application of Salvia miltiorrhiza in the treatment of gastric cancer.

**Reviewer #1: 2)Secondly，this article states that factors derived from Salvia miltiorrhiza can regulate gene expression and inhibit the progression of gastric cancer. However, the article does not provide a specific discussion of this argument. It is recommended that the author elaborate in detail on the possible mechanisms involved.**

**Respond to Reviewer :** Thank you for your valuable advice. In response to your comments, we have made corresponding revisions to the manuscript. The specific adjustments are as follows:

In the revised manuscript, we have further elaborated that miRNAs (such as miR4102) contained in Danshen-derived ELNs can target and regulate the key cell cycle gene CDK2, thereby inhibiting tumor cell proliferation. These miRNAs also activate signaling pathways such as FoxO and p53, leading to cell cycle arrest and apoptosis. Additionally, they may suppress NF-κB-mediated inflammation(a hallmark of adipocytokine signaling dysregulation), improve the tumor microenvironment, and exert anticancer effects through multiple mechanisms.

**Reviewer #2:**

**Introduction**
**The introduction should focus on the background information relevant to the study, rather than presenting results or discussing the methods, which should be reserved for their respective sections. It is important to elaborate on the properties of Salvia miltiorrhiza Bunge that make it a promising candidate for cancer treatment. Additionally, the bioactive components of this plant, such as Salvianolic acids and Tanshinones (with a particular emphasis on Tanshinone IIA), should be discussed in relation to their potential therapeutic effects in cancer therapy.**
**Respond to Reviewer :** Thank you very much for your valuable comments on our manuscript. We have revised the Introduction section according to your suggestions. Specifically, we have: (1) removed content related to our study’s results and methods to ensure the introduction focuses on background information; (2) elaborated on the properties of Salvia miltiorrhiza Bunge that make it a promising candidate for cancer treatment; and (3) added information about its major bioactive components, such as Salvianolic acids and Tanshinones (with particular emphasis on Tanshinone IIA), and their potential therapeutic effects in cancer therapy. We hope these revisions improve the scientific rigor and clarity of our manuscript.

**Materials and Methods**
**（A）In the cell culture section, please specify the name and brand of the culture media used.**

**Respond to Reviewer :** Thank you for your valuable feedback. We appreciate your attention to detail and have carefully revised the Materials and Methods section as suggested. The specific name and brand of the culture medium (DMEM ,Procell, China) have now been explicitly stated in the revised text.

**(B) Did your study utilize all parts of the plant for the isolation and purification of exosome-like nanoparticles? Please clarify this detail.**

**Respond to Reviewer :** We sincerely thank the editor for the valuable comment. In our study, only the root part of Salvia miltiorrhiza (Danshen) was used for the isolation and purification of exosome-like nanoparticles (ELNs). We have revised the relevant section in the manuscript to clarify this detail.

**(C) For the cell viability assays, the number of cells seeded in each well of the 96-well plate should be included. Additionally, please specify the cell count present in 200 μL of HGC-27.**

**Respond to Reviewer :** We sincerely thank the editor for the valuable comments. In accordance with your suggestion, we have now specified the number of HGC-27 cells seeded per well in the 96-well plate. Specifically, 5,000 cells were seeded in 200 μL of medium per well. The revised information can be found in the Methods section (Cell viability detection).

**(D) In the wound healing assay, it is necessary to report the cell count in each well of the 6-well plate. Furthermore, please include the formula used to estimate the migration rate. Mention the software utilized for analysis, such as ImageJ, T-scratch, or any other relevant tools.**

**Respond to Reviewer :** Thank you very much for your valuable comments and suggestions. In response, we have added the specific cell count (2×10^5^ cells per well) used in the 6-well plates for the wound healing assay. We have also included the formula used to calculate the migration rate and specified that ImageJ software was utilized for the analysis of wound area. We sincerely appreciate your guidance, which has helped us improve the clarity and rigor of our methods section.

**(E)For the colony formation assay, similarly, please include the number of cells seeded in each well of the 96-well plate. Also, specify the number of cells present in 200 μL of HGC-27.**

**Respond to Reviewer :** Thank you very much for your valuable comments and suggestions. According to your advice, we have revised the Methods section to specify the number of HGC-27 cells seeded in each well of the six-well and 96-well plates. We have also clarified the number of cells present in 200 μL of culture medium. The revised text now reads as follows:

“For the colony formation assay, HGC-27 cells were seeded at a density of 500 cells per well in six-well plates and incubated for 10–14 days. After incubation, the colonies were fixed and stained with 0.4% crystal violet (Solarbio, Beijing, China). The number of colonies was counted using an inverted microscope (Leica, Germany). For the 96-well plate assay, 1,000 HGC-27 cells were seeded per well in 200 μL of culture medium.”

**Results**
**（A）Please clarify why a particle concentration of 10^9^ particles/mL was chosen for the experiments. Would it not have been more informative to evaluate the tests with varying particle concentrations in order to assess a dose-response relationship?**

**Respond to Reviewer :** Thank you very much for your valuable suggestion and for pointing out this important aspect of our experimental design.

We selected a particle concentration of 10^9^ particles/mL based on preliminary experiments and relevant literature, which indicated that this concentration is sufficient to elicit a measurable response under our experimental conditions. Our primary aim in this study was to establish a proof-of-concept and to ensure the reproducibility of the observed effects at a concentration commonly used in similar studies.

We fully agree that evaluating a range of particle concentrations to assess a dose-response relationship would provide more comprehensive insights into the system’s behavior. Due to limitations in time and resources, we focused on a single, representative concentration in the current work. However, we recognize the importance of this point and plan to include dose-response experiments in our future studies to further elucidate the relationship between particle concentration and the observed effects.

**(B) In the results section, it is mentioned that OD 450 was used on the vertical axis to assess cell proliferation, whereas OD 490 was cited in the materials and methods section. Please ensure consistency in these values.**

**Respond to Reviewer :** Thank you for your valuable comments on our manuscript. In response to your suggestions, we have made the following amendments:The absorbance wavelength of cell proliferation detection is wrong. We have verified and uniformly adopted 450 nm measurement, and the 490 nm in the material method part is a clerical error, which has been corrected.

**(C) Regarding the** **migration rate, although the text mentions a significant difference between the control group and the group treated with the exosome-like nanoparticles, this difference is not evident in the accompanying image, and the significance level is not indicated on the columns of the graph. Please address this inconsistency.**

**Respond to Reviewer :** Thank you for your valuable comments on our manuscript. In response to your suggestions, we have made the following amendments: In the wound healing experiment, the image is inconsistent with the statistical data. We added the statistical significance label and updated the migration experiment image to ensure the consistency of the graphic content.

**(D) Finally, ensure that the naming conventions for experimental groups are consistent throughout the manuscript. For example, in Figure 3, "EVs," "GELNs," and "DS-EVs" are used interchangeably; please standardize the terminology.
Respond to Reviewer :** Thank you very much for your valuable comments and suggestions. Regarding your advice to ensure consistency in the naming conventions for experimental groups—for example, the interchangeable use of “EVs,” “GELNs,” and “DS-EVs” in Figure 3—we have carefully reviewed and revised the entire manuscript, including all figures, figure legends, and the main text. We have now standardized the terminology and consistently use “DS-EVs” to refer to the relevant experimental group throughout the manuscript. The term is clearly defined at its first appearance to avoid any confusion for readers.

**Discussion**
The results presented should be critically analyzed and compared with findings from other studies to provide context and a deeper understanding of the significance of the findings. Rather than merely presenting your own data, it is important to juxtapose your results with those of similar investigations to highlight consistencies, discrepancies, and potential reasons for these differences.

**Respond to Reviewer :** Thank you for your valuable comments on our manuscript. In response to your suggestions, we have made the following amendments:

Thank you very much for your valuable comments and suggestions. In response to your advice, we have revised the manuscript to provide a more critical analysis of our results and have compared our findings with those from other relevant studies. Specifically, we have discussed the similarities and differences between our results and previous reports on plant-derived ELNs, and have explored possible reasons for these observations. We believe these revisions have strengthened the context and significance of our findings.
