# Supplementary material for: Characterization and functional analysis of miRNAs in Salvia miltiorrhiza-isolated exosome-like nanoparticles for gastric cancer treatment
Source: PLoS One. 2026 Jul 16;21(7):e0353354. doi: 10.1371/journal.pone.0353354 (PMC13375030; doi:10.1371/journal.pone.0353354)
Supplement: Attachment — Submitted filename: Reviewer_Report_PONE-D-24-54868R1.docx [file pone.0353354.s012.docx]

Dear Editor,

I have carefully reviewed the revised manuscript by the authors (PONED2454868R1) and their detailed responses to the comments made by the two reviewers. Based on my assessment, I believe the authors have comprehensively and satisfactorily addressed all the issues. Here is my detailed evaluation.

1. Regarding Reviewer 1’s comments

The authors have appropriately acknowledged the limitation that largesample, multicenter clinical studies are still lacking for Danshen in gastric cancer treatment, and they have indicated their intention to pursue such studies in the future. More importantly, they have substantially expanded the discussion on the possible molecular mechanisms, including the roles of miR4102 targeting CDK2, activation of FoxO/p53 pathways, and suppression of NFκB‑mediated inflammation. These revisions have directly addressed the request for a more detailed mechanistic explanation. I find the response fully adequate.

2. Regarding Reviewer 2’s comments

The authors have carefully followed each suggestion:

The Introduction has been refocused on background information, with added details on the anticancer properties of Salvia miltiorrhiza and its active components (salvianolic acids, tanshinones, especially tanshinone IIA).

Materials and Methods now include the specific culture medium brand, clarification that only the root part was used, exact cell seeding numbers, the wound healing formula, and the software used (ImageJ).

Results provide a reasonable justification for using a single particle concentration, with acknowledgment of the value of future dose‑response studies. The OD wavelength has been corrected, the wound healing images and significance labels have been updated, and the terminology has been standardized to “DS‑EVs” throughout.

Discussion has been revised to critically compare the findings with other studies on plant‑derived ELNs, which significantly improves the contextual depth.

All these revisions are appropriate and have enhanced the clarity and rigor of the manuscript.

3. Additional observations and recommendation

In conclusion, the authors have responded comprehensively and professionally to all issues raised. The manuscript is now much improved and, in my opinion, meets the standards for publication in PLOS ONE.

Therefore, I recommend acceptance of the manuscript in its current form.

Thank you for the opportunity to review this work.

Sincerely,
