# Supplementary material for: Characterization and functional analysis of miRNAs in Salvia miltiorrhiza-isolated exosome-like nanoparticles for gastric cancer treatment
Source: PLoS One. 2026 Jul 16;21(7):e0353354. doi: 10.1371/journal.pone.0353354 (PMC13375030; doi:10.1371/journal.pone.0353354)
Supplement: Peer review history

# Peer review: Decision Letter 0

**DOI:** 10.1371/journal.pone.0353354.r001

**Authors:** Minhui Li

9 Apr 2025

PONE-D-24-54868

Characterization and Functional Analysis of miRNAs in Salvia miltiorrhiza-Isolated Exosome-Like Nanoparticles for Gastric Cancer Treatment

PLOS ONE

Dear Dr. Chen,

Thank you for submitting your manuscript to PLOS ONE. After careful consideration, we feel that it has merit but does not fully meet PLOS ONE’s publication criteria as it currently stands. Therefore, we invite you to submit a revised version of the manuscript that addresses the points raised during the review process.

Please submit your revised manuscript by May 24 2025 11:59PM. If you will need more time than this to complete your revisions, please reply to this message or contact the journal office at plosone@plos.org. When you're ready to submit your revision, log on to https://www.editorialmanager.com/pone/ and select the 'Submissions Needing Revision' folder to locate your manuscript file.

Please include the following items when submitting your revised manuscript:

- A rebuttal letter that responds to each point raised by the academic editor and reviewer(s). You should upload this letter as a separate file labeled 'Response to Reviewers'.
- A marked-up copy of your manuscript that highlights changes made to the original version. You should upload this as a separate file labeled 'Revised Manuscript with Track Changes'.
- An unmarked version of your revised paper without tracked changes. You should upload this as a separate file labeled 'Manuscript'.

If you would like to make changes to your financial disclosure, please include your updated statement in your cover letter. Guidelines for resubmitting your figure files are available below the reviewer comments at the end of this letter.

If applicable, we recommend that you deposit your laboratory protocols in protocols.io to enhance the reproducibility of your results. Protocols.io assigns your protocol its own identifier (DOI) so that it can be cited independently in the future. For instructions see: https://journals.plos.org/plosone/s/submission-guidelines#loc-laboratory-protocols. Additionally, PLOS ONE offers an option for publishing peer-reviewed Lab Protocol articles, which describe protocols hosted on protocols.io. Read more information on sharing protocols at https://plos.org/protocols?utm_medium=editorial-email&utm_source=authorletters&utm_campaign=protocols.

We look forward to receiving your revised manuscript.

Kind regards,

Minhui Li, PhD

Academic Editor

PLOS ONE

Journal Requirements:

When submitting your revision, we need you to address these additional requirements.

1. Please ensure that your manuscript meets PLOS ONE's style requirements, including those for file naming. The PLOS ONE style templates can be found at

https://journals.plos.org/plosone/s/file?id=wjVg/PLOSOne_formatting_sample_main_body.pdf and

https://journals.plos.org/plosone/s/file?id=ba62/PLOSOne_formatting_sample_title_authors_affiliations.pdf.

2. PLOS requires an ORCID iD for the corresponding author in Editorial Manager on papers submitted after December 6th, 2016. Please ensure that you have an ORCID iD and that it is validated in Editorial Manager. To do this, go to ‘Update my Information’ (in the upper left-hand corner of the main menu), and click on the Fetch/Validate link next to the ORCID field. This will take you to the ORCID site and allow you to create a new iD or authenticate a pre-existing iD in Editorial Manager.

3. Thank you for stating the following in the Acknowledgments Section of your manuscript:

“This work was financially supported by the Shaoxing city Keqiao District Hospital of Traditional Chinese Medicine”

We note that you have provided additional information within the Acknowledgements Section that is not currently declared in your Funding Statement. Please note that funding information should not appear in the Acknowledgments section or other areas of your manuscript. We will only publish funding information present in the Funding Statement section of the online submission form.

Please remove any funding-related text from the manuscript and let us know how you would like to update your Funding Statement. Currently, your Funding Statement reads as follows:

“The author(s) received no specific funding for this work.”

Please include your amended statements within your cover letter; we will change the online submission form on your behalf.

[Note: HTML markup is below. Please do not edit.]

Reviewers' comments:

Reviewer's Responses to Questions

**Comments to the Author**

1. Is the manuscript technically sound, and do the data support the conclusions?

The manuscript must describe a technically sound piece of scientific research with data that supports the conclusions. Experiments must have been conducted rigorously, with appropriate controls, replication, and sample sizes. The conclusions must be drawn appropriately based on the data presented.

Reviewer #1: Yes

Reviewer #2: Yes

**********

2. Has the statistical analysis been performed appropriately and rigorously?

Reviewer #1: Yes

Reviewer #2: No

**********

3. Have the authors made all data underlying the findings in their manuscript fully available?

The PLOS Data policy requires authors to make all data underlying the findings described in their manuscript fully available without restriction, with rare exception (please refer to the Data Availability Statement in the manuscript PDF file). The data should be provided as part of the manuscript or its supporting information, or deposited to a public repository. For example, in addition to summary statistics, the data points behind means, medians and variance measures should be available. If there are restrictions on publicly sharing data—e.g. participant privacy or use of data from a third party—those must be specified.

Reviewer #1: Yes

Reviewer #2: Yes

**********

4. Is the manuscript presented in an intelligible fashion and written in standard English?

PLOS ONE does not copyedit accepted manuscripts, so the language in submitted articles must be clear, correct, and unambiguous. Any typographical or grammatical errors should be corrected at revision, so please note any specific errors here.

Reviewer #1: Yes

Reviewer #2: No

**********

5. Review Comments to the Author

Please use the space provided to explain your answers to the questions above. You may also include additional comments for the author, including concerns about dual publication, research ethics, or publication ethics. (Please upload your review as an attachment if it exceeds 20,000 characters)

Reviewer #1: Firstly�multiple active components in Salvia miltiorrhiza have antitumor effects on gastric cancer, such as salvianolic acid B, tanshinone I, tanshinone IIA, etc. They exert their curative effects by regulating multiple molecular targets, such as cell cycle regulatory proteins and apoptosis-related proteins. The anti-gastric cancer effect of Salvia miltiorrhiza has been preliminarily confirmed in clinical and animal experiments. However, due to the lack of large-sample and multi-center clinical studies, its status in the treatment of gastric cancer still needs to be further determined.Secondly�this article states that factors derived from Salvia miltiorrhiza can regulate gene expression and inhibit the progression of gastric cancer.However, the article does not provide a specific discussion of this argument. It is recommended that the author elaborate in detail on the possible mechanisms involved.

Reviewer #2: Introduction

The introduction should focus on the background information relevant to the study, rather than presenting results or discussing the methods, which should be reserved for their respective sections. It is important to elaborate on the properties of Salvia miltiorrhiza Bunge that make it a promising candidate for cancer treatment. Additionally, the bioactive components of this plant, such as Salvianolic acids and Tanshinones (with a particular emphasis on Tanshinone IIA), should be discussed in relation to their potential therapeutic effects in cancer therapy.

Materials and Methods

In the cell culture section, please specify the name and brand of the culture media used.

Did your study utilize all parts of the plant for the isolation and purification of exosome-like nanoparticles? Please clarify this detail.

For the cell viability assays, the number of cells seeded in each well of the 96-well plate should be included. Additionally, please specify the cell count present in 200 μL of HGC-27.

In the wound healing assay, it is necessary to report the cell count in each well of the 6-well plate. Furthermore, please include the formula used to estimate the migration rate. Mention the software utilized for analysis, such as ImageJ, T-scratch, or any other relevant tools.

For the colony formation assay, similarly, please include the number of cells seeded in each well of the 96-well plate. Also, specify the number of cells present in 200 μL of HGC-27.

Results

Please clarify why a particle concentration of 109 particles/mL was chosen for the experiments. Would it not have been more informative to evaluate the tests with varying particle concentrations in order to assess a dose-response relationship?

In the results section, it is mentioned that OD 450 was used on the vertical axis to assess cell proliferation, whereas OD 490 was cited in the materials and methods section. Please ensure consistency in these values.

Regarding the migration rate, although the text mentions a significant difference between the control group and the group treated with the exosome-like nanoparticles, this difference is not evident in the accompanying image, and the significance level is not indicated on the columns of the graph. Please address this inconsistency.

Finally, ensure that the naming conventions for experimental groups are consistent throughout the manuscript. For example, in Figure 3, "EVs," "GELNs," and "DS-EVs" are used interchangeably; please standardize the terminology.

Discussion

The results presented should be critically analyzed and compared with findings from other studies to provide context and a deeper understanding of the significance of the findings. Rather than merely presenting your own data, it is important to juxtapose your results with those of similar investigations to highlight consistencies, discrepancies, and potential reasons for these differences.

**********

6. PLOS authors have the option to publish the peer review history of their article (what does this mean?). If published, this will include your full peer review and any attached files.

If you choose “no”, your identity will remain anonymous but your review may still be made public.

**Do you want your identity to be public for this peer review?**  For information about this choice, including consent withdrawal, please see our Privacy Policy.

Reviewer #1: No

Reviewer #2: No

**********

[NOTE: If reviewer comments were submitted as an attachment file, they will be attached to this email and accessible via the submission site. Please log into your account, locate the manuscript record, and check for the action link "View Attachments". If this link does not appear, there are no attachment files.]

While revising your submission, please upload your figure files to the Preflight Analysis and Conversion Engine (PACE) digital diagnostic tool, https://pacev2.apexcovantage.com/. PACE helps ensure that figures meet PLOS requirements. To use PACE, you must first register as a user. Registration is free. Then, login and navigate to the UPLOAD tab, where you will find detailed instructions on how to use the tool. If you encounter any issues or have any questions when using PACE, please email PLOS at figures@plos.org. Please note that Supporting Information files do not need this step.

# Author comment: Author response to Decision Letter 1

**DOI:** 10.1371/journal.pone.0353354.r002

4 Jun 2025

Characterization and Functional Analysis of miRNAs in Salvia miltiorrhiza-Isolated Exosome-Like Nanoparticles for Gastric Cancer Treatment

Review Comments to the Author

Please use the space provided to explain your answers to the questions above. You may also include additional comments for the author, including concerns about dual publication, research ethics, or publication ethics. (Please upload your review as an attachment if it exceeds 20,000 characters).

Reviewer #1: Firstly�multiple active components in Salvia miltiorrhiza have antitumor effects on gastric cancer, such as salvianolic acid B, tanshinone I, tanshinone IIA, etc. They exert their curative effects by regulating multiple molecular targets, such as cell cycle regulatory proteins and apoptosis-related proteins. The anti-gastric cancer effect of Salvia miltiorrhiza has been preliminarily confirmed in clinical and animal experiments. However, due to the lack of large-sample and multi-center clinical studies, its status in the treatment of gastric cancer still needs to be further determined.

Respond to Reviewer :Thank you very much for your careful review of our manuscript and your valuable comments. We fully agree with your opinion regarding the antitumor effects of multiple active components in Salvia miltiorrhiza on gastric cancer. As you pointed out, although several clinical and animal studies have preliminarily confirmed that Salvia miltiorrhiza and its active components (such as salvianolic acid B, tanshinone I, and tanshinone IIA) exert antitumor effects by regulating multiple molecular targets, including cell cycle regulatory proteins and apoptosis-related proteins, the exact status of Salvia miltiorrhiza in the treatment of gastric cancer still needs to be further determined due to the lack of large-sample and multi-center clinical studies.

We sincerely appreciate your professional suggestions, which are of great significance for guiding our future research. In our subsequent work, we will pay close attention to and actively promote large-scale, multi-center clinical studies in this field, in order to provide more solid evidence for the application of Salvia miltiorrhiza in the treatment of gastric cancer.

Secondly�this article states that factors derived from Salvia miltiorrhiza can regulate gene expression and inhibit the progression of gastric cancer. However, the article does not provide a specific discussion of this argument. It is recommended that the author elaborate in detail on the possible mechanisms involved.

Respond to Reviewer : Thank you for your valuable advice. In response to your comments, we have made corresponding revisions to the manuscript. The specific adjustments are as follows:

In the revised manuscript, we have further elaborated that miRNAs (such as miR4102) contained in Danshen-derived ELNs can target and regulate the key cell cycle gene CDK2, thereby inhibiting tumor cell proliferation. These miRNAs also activate signaling pathways such as FoxO and p53, leading to cell cycle arrest and apoptosis. Additionally, they may suppress NF-κB-mediated inflammation(a hallmark of adipocytokine signaling dysregulation), improve the tumor microenvironment, and exert anticancer effects through multiple mechanisms.

Reviewer #2:

Introduction

The introduction should focus on the background information relevant to the study, rather than presenting results or discussing the methods, which should be reserved for their respective sections. It is important to elaborate on the properties of Salvia miltiorrhiza Bunge that make it a promising candidate for cancer treatment. Additionally, the bioactive components of this plant, such as Salvianolic acids and Tanshinones (with a particular emphasis on Tanshinone IIA), should be discussed in relation to their potential therapeutic effects in cancer therapy.

Respond to Reviewer : Thank you very much for your valuable comments on our manuscript. We have revised the Introduction section according to your suggestions. Specifically, we have: (1) removed content related to our study’s results and methods to ensure the introduction focuses on background information; (2) elaborated on the properties of Salvia miltiorrhiza Bunge that make it a promising candidate for cancer treatment; and (3) added information about its major bioactive components, such as Salvianolic acids and Tanshinones (with particular emphasis on Tanshinone IIA), and their potential therapeutic effects in cancer therapy. We hope these revisions improve the scientific rigor and clarity of our manuscript.

Materials and Methods

�A�In the cell culture section, please specify the name and brand of the culture media used.

Respond to Reviewer : Thank you for your valuable feedback. We appreciate your attention to detail and have carefully revised the Materials and Methods section as suggested. The specific name and brand of the culture medium (DMEM ,Procell, China) have now been explicitly stated in the revised text.

(B) Did your study utilize all parts of the plant for the isolation and purification of exosome-like nanoparticles? Please clarify this detail.

Respond to Reviewer : We sincerely thank the editor for the valuable comment. In our study, only the root part of Salvia miltiorrhiza (Danshen) was used for the isolation and purification of exosome-like nanoparticles (ELNs). We have revised the relevant section in the manuscript to clarify this detail.

(C) For the cell viability assays, the number of cells seeded in each well of the 96-well plate should be included. Additionally, please specify the cell count present in 200 μL of HGC-27.

Respond to Reviewer : We sincerely thank the editor for the valuable comments. In accordance with your suggestion, we have now specified the number of HGC-27 cells seeded per well in the 96-well plate. Specifically, 5,000 cells were seeded in 200 μL of medium per well. The revised information can be found in the Methods section (Cell viability detection).

(D) In the wound healing assay, it is necessary to report the cell count in each well of the 6-well plate. Furthermore, please include the formula used to estimate the migration rate. Mention the software utilized for analysis, such as ImageJ, T-scratch, or any other relevant tools.

Respond to Reviewer : Thank you very much for your valuable comments and suggestions. In response, we have added the specific cell count (2×105 cells per well) used in the 6-well plates for the wound healing assay. We have also included the formula used to calculate the migration rate and specified that ImageJ software was utilized for the analysis of wound area. We sincerely appreciate your guidance, which has helped us improve the clarity and rigor of our methods section.

(E)For the colony formation assay, similarly, please include the number of cells seeded in each well of the 96-well plate. Also, specify the number of cells present in 200 μL of HGC-27.

Respond to Reviewer : Thank you very much for your valuable comments and suggestions. According to your advice, we have revised the Methods section to specify the number of HGC-27 cells seeded in each well of the six-well and 96-well plates. We have also clarified the number of cells present in 200 μL of culture medium. The revised text now reads as follows:

“For the colony formation assay, HGC-27 cells were seeded at a density of 500 cells per well in six-well plates and incubated for 10–14 days. After incubation, the colonies were fixed and stained with 0.4% crystal violet (Solarbio, Beijing, China). The number of colonies was counted using an inverted microscope (Leica, Germany). For the 96-well plate assay, 1,000 HGC-27 cells were seeded per well in 200 μL of culture medium.”

Results

�A�Please clarify why a particle concentration of 109 particles/mL was chosen for the experiments. Would it not have been more informative to evaluate the tests with varying particle concentrations in order to assess a dose-response relationship?

Respond to Reviewer : Thank you very much for your valuable suggestion and for pointing out this important aspect of our experimental design.

We selected a particle concentration of 109 particles/mL based on preliminary experiments and relevant literature, which indicated that this concentration is sufficient to elicit a measurable response under our experimental conditions. Our primary aim in this study was to establish a proof-of-concept and to ensure the reproducibility of the observed effects at a concentration commonly used in similar studies.

We fully agree that evaluating a range of particle concentrations to assess a dose-response relationship would provide more comprehensive insights into the system’s behavior. Due to limitations in time and resources, we focused on a single, representative concentration in the current work. However, we recognize the importance of this point and plan to include dose-response experiments in our future studies to further elucidate the relationship between particle concentration and the observed effects.

(B) In the results section, it is mentioned that OD 450 was used on the vertical axis to assess cell proliferation, whereas OD 490 was cited in the materials and methods section. Please ensure consistency in these values.

Respond to Reviewer : Thank you for your valuable comments on our manuscript. In response to your suggestions, we have made the following amendments:The absorbance wavelength of cell proliferation detection is wrong. We have verified and uniformly adopted 450 nm measurement, and the 490 nm in the material method part is a clerical error, which has been corrected.

(C) Regarding the migration rate, although the text mentions a significant difference between the control group and the group treated with the exosome-like nanoparticles, this difference is not evident in the accompanying image, and the significance level is not indicated on the columns of the graph. Please address this inconsistency.

Respond to Reviewer : Thank you for your valuable comments on our manuscript. In response to your suggestions, we have made the following amendments: In the wound healing experiment, the image is inconsistent with the statistical data. We added the statistical significance label and updated the migration experiment image to ensure the consistency of the graphic content.

(D) Finally, ensure that the naming conventions for experimental groups are consistent throughout the manuscript. For example, in Figure 3, "EVs," "GELNs," and "DS-EVs" are used interchangeably; please standardize the terminology.

Respond to Reviewer : Thank you very much for your valuable comments and suggestions. Regarding your advice to ensure consistency in the naming conventions for experimental groups—for example, the interchangeable use of “EVs,” “GELNs,” and “DS-EVs” in Figure 3—we have carefully reviewed and revised the entire manuscript, including all figures, figure legends, and the main text. We have now standardized the terminology and consistently use “DS-EVs” to refer to the relevant experimental group throughout the manuscript. The term is clearly defined at its first appearance to avoid any confusion for readers.

Discussion

The results presented should be critically analyzed and compared with findings from other studies to provide context and a deeper understanding of the significance of the findings. Rather than merely presenting your own data, it is important to juxtapose your results with those of similar investigations to highlight consistencies, discrepancies, and potential reasons for these differences.

Respond to Reviewer : Thank you for your valuable comments on our manuscript. In response to your suggestions, we have made the following amendments:

Thank you very much for your valuable comments and suggestions. In response to your advice, we have revised the manuscript to provide a more critical analysis of our results and have compared our findings with those from other relevant studies. Specifically, we have discussed the similarities and differences between our results and previous reports on plant-derived ELNs, and have explored possible reasons for these observations. We believe these revisions have strengthened the context and significance of our findings.

Attachment Submitted filename: Response to Reviewers.docx

# Peer review: Decision Letter 1

**DOI:** 10.1371/journal.pone.0353354.r003

**Authors:** Minhui Li

23 Jun 2026

Characterization and Functional Analysis of miRNAs in Salvia miltiorrhiza-Isolated Exosome-Like Nanoparticles for Gastric Cancer Treatment

PONE-D-24-54868R1

Dear Dr.  Gang Chen,

We’re pleased to inform you that your manuscript has been judged scientifically suitable for publication and will be formally accepted for publication once it meets all outstanding technical requirements.

Within one week, you’ll receive an e-mail detailing the required amendments. When these have been addressed, you’ll receive a formal acceptance letter and your manuscript will be scheduled for publication.

An invoice will be generated when your article is formally accepted. Please note, if your institution has a publishing partnership with PLOS and your article meets the relevant criteria, all or part of your publication costs will be covered. Please make sure your user information is up-to-date by logging into Editorial Manager at Editorial Manager® and clicking the ‘Update My Information' link at the top of the page. For questions related to billing, please contact billing support.

If your institution or institutions have a press office, please notify them about your upcoming paper to help maximize its impact. If they’ll be preparing press materials, please inform our press team as soon as possible -- no later than 48 hours after receiving the formal acceptance. Your manuscript will remain under strict press embargo until 2 pm Eastern Time on the date of publication. For more information, please contact onepress@plos.org.

Kind regards,

Minhui Li, PhD

Academic Editor

PLOS One

Additional Editor Comments (optional):

Reviewers' comments:

Reviewer's Responses to Questions

**Comments to the Author**

1. If the authors have adequately addressed your comments raised in a previous round of review and you feel that this manuscript is now acceptable for publication, you may indicate that here to bypass the “Comments to the Author” section, enter your conflict of interest statement in the “Confidential to Editor” section, and submit your "Accept" recommendation.

Reviewer #1: All comments have been addressed

Reviewer #3: (No Response)

**********

2. Is the manuscript technically sound, and do the data support the conclusions?

The manuscript must describe a technically sound piece of scientific research with data that supports the conclusions. Experiments must have been conducted rigorously, with appropriate controls, replication, and sample sizes. The conclusions must be drawn appropriately based on the data presented.

Reviewer #1: Yes

Reviewer #3: (No Response)

**********

3. Has the statistical analysis been performed appropriately and rigorously?

Reviewer #1: Yes

Reviewer #3: (No Response)

**********

4. Have the authors made all data underlying the findings in their manuscript fully available?

The PLOS Data policy requires authors to make all data underlying the findings described in their manuscript fully available without restriction, with rare exception (please refer to the Data Availability Statement in the manuscript PDF file). The data should be provided as part of the manuscript or its supporting information, or deposited to a public repository. For example, in addition to summary statistics, the data points behind means, medians and variance measures should be available. If there are restrictions on publicly sharing data—e.g. participant privacy or use of data from a third party—those must be specified.

Reviewer #1: Yes

Reviewer #3: (No Response)

**********

5. Is the manuscript presented in an intelligible fashion and written in standard English?

PLOS ONE does not copyedit accepted manuscripts, so the language in submitted articles must be clear, correct, and unambiguous. Any typographical or grammatical errors should be corrected at revision, so please note any specific errors here.

Reviewer #1: Yes

Reviewer #3: (No Response)

**********

6. Review Comments to the Author

Please use the space provided to explain your answers to the questions above. You may also include additional comments for the author, including concerns about dual publication, research ethics, or publication ethics. (Please upload your review as an attachment if it exceeds 20,000 characters)

Reviewer #1: The author has revised the previous issue, the article is in line with reality, the data supports the conclusion, and appropriate statistical analysis has been performed.

Reviewer #3: (No Response)

**********

7. PLOS authors have the option to publish the peer review history of their article (what does this mean?). If published, this will include your full peer review and any attached files.

If you choose “no”, your identity will remain anonymous but your review may still be made public.

**Do you want your identity to be public for this peer review?**  For information about this choice, including consent withdrawal, please see our Privacy Policy.

Reviewer #1: No

Reviewer #3: No

**********

Attachment Submitted filename: Reviewer_Report_PONE-D-24-54868R1.docx

# Editor report: Acceptance letter

**DOI:** 10.1371/journal.pone.0353354.r004

**Authors:** Minhui Li

PONE-D-24-54868R1

PLOS One

Dear Dr. Chen,

I'm pleased to inform you that your manuscript has been deemed suitable for publication in PLOS One. Congratulations! Your manuscript is now being handed over to our production team.

At this stage, our production department will prepare your paper for publication. This includes ensuring the following:

* All references, tables, and figures are properly cited

* All relevant supporting information is included in the manuscript submission,

* There are no issues that prevent the paper from being properly typeset

You will receive further instructions from the production team, including instructions on how to review your proof when it is ready. Please keep in mind that we are working through a large volume of accepted articles, so please give us a few days to review your paper and let you know the next and final steps.

Lastly, if your institution or institutions have a press office, please let them know about your upcoming paper now to help maximize its impact. If they'll be preparing press materials, please inform our press team within the next 48 hours. Your manuscript will remain under strict press embargo until 2 pm Eastern Time on the date of publication. For more information, please contact onepress@plos.org.

You will receive an invoice from PLOS for your publication fee after your manuscript has reached the completed accept phase. If you receive an email requesting payment before acceptance or for any other service, this may be a phishing scheme. Learn how to identify phishing emails and protect your accounts at https://explore.plos.org/phishing.

If we can help with anything else, please email us at customercare@plos.org.

Thank you for submitting your work to PLOS ONE and supporting open access.

Kind regards,

PLOS ONE Editorial Office Staff

on behalf of

Dr. Minhui Li

Academic Editor

PLOS One
